# Is Brukinsa (Zanubrutinib) a Safer Bruton’s Tyrosine Kinase (BTK) Inhibitor in Relapsed or Refractory Chronic Lymphocytic Leukemia? A Systematic Review and Meta-Analysis

**DOI:** 10.3390/ph19030467

**Published:** 2026-03-12

**Authors:** Helal F. Hetta, Ayman Salama, Turki A. Aljuaid, Yazan T. Mojmami, Riyadh S. Alotibi, Ahmed M. Alqabaly, Nawaf A. Aldosari, Sami A. Alshahri, Walid I. A. Asiri, Raed S. Alamri, Fayez A. Alanazi, Malek S. A. Alenazi, Mohammed H. Albuhayri, Yasmin N. Ramadan, Reem Sayad

**Affiliations:** 1 Division of Microbiology, Immunology and Biotechnology, Department of Natural Products and Alternative Medicine, Faculty of Pharmacy, University of Tabuk, Tabuk 71491, Saudi Arabia; 2 Department of Pharmaceutics, Faculty of Pharmacy, University of Tabuk, Tabuk 71491, Saudi Arabia; agrawan@ut.edu.sa; 3 PharmD Program, Faculty of Pharmacy, University of Tabuk, Tabuk 71491, Saudi Arabia; 441003757@stu.ut.edu.sa (T.A.A.); 441008409@stu.ut.edu.sa (Y.T.M.); 441008505@stu.ut.edu.sa (R.S.A.); 441008507@stu.ut.edu.sa (A.M.A.); 441004103@stu.ut.edu.sa (N.A.A.); 431006437@stu.ut.edu.sa (S.A.A.); 441001126@stu.ut.edu.sa (W.I.A.A.); 441003569@stu.ut.edu.sa (R.S.A.); 441008370@stu.ut.edu.sa (F.A.A.); 441007391@stu.ut.edu.sa (M.S.A.A.); 431001364@stu.ut.edu.sa (M.H.A.); 4 Department of Microbiology and Immunology, Faculty of Pharmacy, Assiut University, Assiut 71515, Egypt; yasmine_mohamed@pharm.aun.edu.eg; 5 Department of Histology, Faculty of Medicine, Assiut University, Assiut 71515, Egypt; reem.17289806@med.aun.edu.eg

**Keywords:** zanubrutinib, BGB-3111, Bruton’s tyrosine kinase inhibitor, chronic lymphocytic leukemia, Brukinsa, small lymphocytic lymphoma, relapsed/refractory

## Abstract

**Background/Objectives:** Zanubrutinib (Brukinsa) is a next-generation Bruton’s tyrosine kinase inhibitor approved for the treatment of relapsed or refractory (R/R) chronic lymphocytic leukemia (CLL) and small lymphocytic lymphoma (SLL); however, a comprehensive quantitative assessment of its safety profile remains limited. **Methods:** A systematic search of PubMed, Scopus, Web of Science, and MEDLINE was conducted to identify clinical trials published up to August 2025 that reported treatment-emergent adverse events (TEAEs) associated with zanubrutinib in patients with R/R CLL/SLL. Pooled incidence estimates were calculated using a random-effects model (DerSimonian and Laird method). **Results:** Four studies comprising 508 patients were included, with median follow-up durations ranging from 15.1 to 34.5 months. The pooled incidence of any-grade adverse events was 98.5% (95% CI, 97.1–99.9), while grade ≥3 adverse events occurred in 67.0% (95% CI, 55.4–78.7). Serious adverse events were reported in 32.2% of patients (95% CI, 25.1–39.3), treatment discontinuation due to toxicity occurred in 7.2% (95% CI, 2.5–11.8), and adverse event-related mortality was observed in 7.1% (95% CI, 0.2–13.9). The most frequently reported hematological adverse events were neutropenia (32.1%) and anemia (26.7%), while common non-hematological adverse events included bleeding events (51.9%), upper respiratory tract infections (27.2%), pneumonia (19.4%), and hypertension (16.4%). Atrial fibrillation occurred in 2.9% of patients. **Conclusions:** Zanubrutinib was associated with a high incidence of adverse events, although rates of treatment discontinuation and atrial fibrillation were relatively low, supporting its tolerability in R/R CLL/SLL within clinical trial settings while highlighting the need for continued long-term and real-world safety monitoring.

## 1. Introduction

The most prevalent type of leukemia globally is small lymphocytic lymphoma (SLL) or chronic lymphocytic leukemia (CLL) [1,2]. Bruton’s tyrosine kinase (BTK) inhibitors exert their therapeutic effects by irreversibly binding to the Cys481 residue within the ATP-binding pocket of BTK, thereby blocking B-cell receptor signaling that promotes malignant B-cell survival. BTK inhibitors have completely changed how CLL/SLL is treated in recent years [1]. Although ibrutinib [3], a first-generation BTK inhibitor, was a breakthrough in the treatment of CLL/SLL, it also exhibits substantial off-target inhibition of other kinases, including TEC family kinases, EGFR, ITK, and Src family kinases, and has been associated with adverse effects such as hematological adverse events (such as reduced hemoglobin, platelet, and/or neutrophil levels) [4], non-hematological AEs such as cardiovascular toxicities (e.g., atrial fibrillation/flutter) [2,5], and hypertension [3,4,6]. Off-target inhibition of other kinases is probably the cause of these effects [1].

In contrast, second-generation BTK inhibitors such as zanubrutinib were developed with the goal of improving kinase selectivity and limiting off-target interactions. Zanubrutinib demonstrates enhanced specificity for BTK relative to many off-target kinases, leading to a favorable safety profile with lower reported rates of cardiovascular toxicities, including atrial fibrillation, compared with first-generation agents [7].

Zanubrutinib is a powerful and specific next-generation BTK inhibitor [7] that has been licensed for the treatment of CLL in China, the United States, and the European Union. It has also been approved for the treatment of SLL in China and the United States (the European Medicines Agency considers SLL to be included in CLL) [8,9,10]. The goal of zanubrutinib’s design was to reduce off-target kinase binding and related adverse events while increasing BTK occupancy [7]. In vitro kinome profiling revealed that zanubrutinib is more selective against BTK than ibrutinib and acalabrutinib [7,11]. The zanubrutinib group exhibited better progression-free survival (PFS), fewer adverse events (AEs) that resulted in therapy discontinuation (15.4% vs. 22.2%), and fewer cardiac adverse events (21.3% vs. 29.6%) in a randomized, phase III trial including patients with R/R CLL/SLL receiving either ibrutinib or zanubrutinib [12].

To date, no comprehensive meta-analysis has systematically synthesized the available evidence on the safety profile of zanubrutinib in this specific population. A clear and quantitative assessment of its safety outcomes is essential to guide clinical decision-making, support regulatory evaluations, and optimize treatment selection in patients with R/R CLL.

While our previous meta-analysis evaluated the efficacy outcomes of zanubrutinib in patients with CLL/SLL [13], the present study builds upon that work by addressing a distinct and clinically important research question focused specifically on safety outcomes. Accordingly, this systematic review and meta-analysis aims to comprehensively evaluate the safety profile of zanubrutinib (Brukinsa) in patients with R/R CLL/SLL. The primary objective is to quantitatively estimate the pooled incidence rates of treatment-emergent adverse events (TEAEs), grade ≥ 3 adverse events, serious adverse events, and treatment discontinuations due to toxicity.

In addition, we aim to identify the most frequently reported adverse events associated with zanubrutinib and to assess the consistency of safety findings across clinical trials while exploring potential sources of heterogeneity. By integrating all available clinical evidence, this study provides a dedicated and robust synthesis of the current data on the safety and tolerability of zanubrutinib, thereby complementing prior efficacy-focused research and supporting informed clinical decision-making.

## 2. Materials and Methods

### 2.1. Study Protocol and Registration

Following the Preferred Reporting Items for Systematic Reviews and Meta-Analyses (PRISMA) [14], we carried out this systematic review and meta-analysis. We followed the recommendations of Cochrane’s Handbook of Systematic Reviews of Interventions [15] at every turn. With the protocol ID CRD420251149417, we registered the protocol in PROSPERO.

### 2.2. Search Strategy and Data Collection

We searched the Web of Science (WoS), Scopus, PubMed, Reaxys, and Medline electronic databases. We searched every study released until August 2025. Details of the search approach are (zanubrutinib OR “BGB-3111”) AND (“chronic lymphocytic leukemia” OR “CLL” OR “small lymphocytic lymphoma” OR “SLL” OR “Leukemia, Lymphocytic, Chronic, B-Cell”).

In two screening processes, we evaluated every study retrieved for our eligibility requirements. Studies that satisfied our eligibility requirements are included. The references to the earlier reviews and the studies we included were manually screened. Two authors completed all screening processes, and a third reviewer settled all conflicts.

### 2.3. Eligibility Criteria

We included trials that fulfilled the illustrated PICO criteria as follows: population (P): patients with refractory or relapsed (R/R) CLL/SLL cases; intervention (I): zanubrutinib; outcome (O): safety outcomes; and study design (S): any clinical trials that assess the safety of zanubrutinib in patients with R/R CLL/SLL.

### 2.4. Data Extraction and Outcome Measurement

Two authors used Excel sheets to extract data. A third author resolved any conflicts during data extraction.

All extracted safety outcomes, including serious adverse events (SAEs), treatment-emergent adverse events (TEAEs), and treatment-related deaths, were harmonized using internationally accepted definitions to ensure consistency across included studies. Safety events were categorized according to the Common Terminology Criteria for Adverse Events (CTCAE) version 5.0 [16], which provides standardized criteria for grading the severity of adverse events in oncology clinical trials, facilitating uniform interpretation of toxicity data across different sources. Additionally, key safety endpoints were aligned with definitions outlined in the International Council for Harmonisation of Technical Requirements for Pharmaceuticals for Human Use (ICH-GCP) E6(R2) guidelines [17], which specify criteria for serious adverse events and clinical trial reporting standards. These harmonization steps were applied during data extraction and coding to ensure that pooled estimates reflect comparable definitions of adverse outcomes across the included trials.

### 2.5. Quality Assessment

The revised Joanna Briggs Institute (JBI) critical appraisal tool for the assessment of risk of bias for RCTs was used by two authors to evaluate the methodology of the included studies [18]. This validated tool evaluates methodological quality across 13 key domains, including randomization, allocation concealment, baseline comparability, blinding of participants, personnel, and outcome assessors, reliability of outcome measures, completeness of follow-up, and appropriate statistical analysis.

The methodological quality of the included non-RCTs was assessed using the Joanna Briggs Institute (JBI) critical appraisal checklist for case series [19]. This validated tool comprises 10 items that evaluate key domains of methodological rigor. Based on the information provided in the publication, each criterion was rated as “Yes” (criterion met), “No” (criterion not met), “Unclear,” or “Not applicable”.

Two independent reviewers performed the appraisal, and discrepancies were resolved by consensus with a third reviewer. The results of the quality appraisal were summarized in tabular form. Notably, studies were not excluded based on quality scores, but the findings of the assessment were considered during data synthesis and interpretation of results.

### 2.6. Data Analysis

We performed a single-arm meta-analysis to estimate the pooled effect of zanubrutinib in patients with CLL, including both treatment-naïve and relapsed/refractory populations. Data synthesis and statistical analyses were conducted using Open Meta-Analyst software V0.24.1.

For each study, we extracted data on the proportion of patients achieving the predefined values. Standard errors were calculated from the reported proportions and sample sizes. When necessary, raw data were used to reconstruct proportions.

A random-effects model (DerSimonian and Laird method) was employed to account for potential clinical and methodological heterogeneity across studies. Pooled estimates were presented with 95% confidence intervals (CIs). Heterogeneity was quantified using Cochran’s Q test (*p* < 0.10 considered significant) and the I^2^ statistic, with thresholds of 25%, 50%, and 75% indicating low, moderate, and high heterogeneity, respectively.

## 3. Results

### 3.1. Study Selection

**Figure 1:**
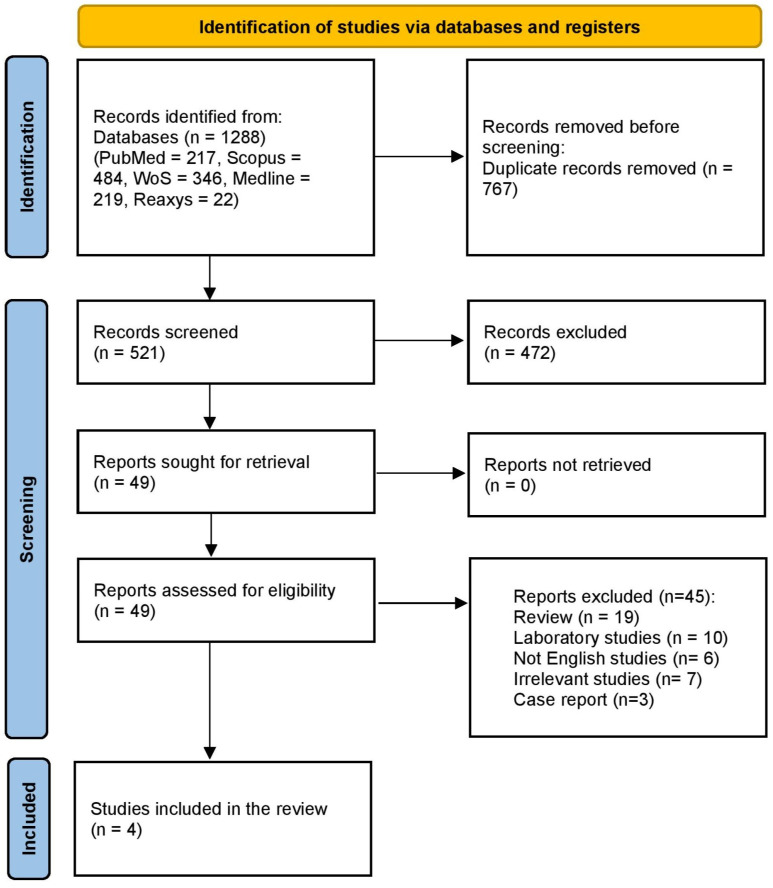
PRISMA flow diagram of the included studies.

Database searches identified 1288 records. After removing duplicates, 521 studies were screened during the title and abstract screening. Full-text screening was conducted for 49 studies, of which 44 were excluded. Ultimately, four studies met the inclusion criteria and were included in this review. More details are presented in Figure 1.

### 3.2. Study Characteristics

Four studies met the eligibility criteria, comprising both clinical trials and real-world observational research [12,20,21,22], encompassing 508 patients treated with zanubrutinib for CLL or SLL. Of these, one was a phase III randomized controlled trial [12], two were phase II single-arm open-label studies [21,22], and one was a phase I/II multicenter open-label trial [20]. Studies were conducted across diverse regions, including the United States, Japan, China, and multiple international centers across North America, Europe, and the Asia-Pacific.

The sample size of patients receiving zanubrutinib ranged from 19 to 327, with follow-up durations varying from 15.1 to 34.5 months (median, 27.9 months). All studies included adult patients (≥18 or ≥20 years) diagnosed with CLL or SLL, with eligibility based on the International Workshop on Chronic Lymphocytic Leukemia (iwCLL) 2008 or 2018 guidelines [23] or equivalent internationally recognized diagnostic criteria. Izutsu et al. (2025) also enrolled patients with other B-cell malignancies, such as Waldenström macroglobulinemia (WM) and mantle cell lymphoma (MCL), for exploratory or safety analyses [20].

Zanubrutinib was administered orally at a dose of 160 mg twice daily in all trials, with some allowing an alternative dosing regimen of 320 mg once daily [21]. Treatment continued until disease progression, unacceptable toxicity, or withdrawal. Across studies, patients were either treatment-naïve (TN) or had R/R disease, with the latter forming the core population for safety assessment in this review.

The phase III ALPINE trial (NCT03734016) provided the largest dataset, including 327 patients randomized to zanubrutinib and followed for a median of 29.6 months [12]. The Japanese phase I/II study (NCT04172246) enrolled 19 patients with CLL/SLL (TN = 14; R/R = 5) and reported a median follow-up of 27.9 months [20]. The phase II U.S. trial (NCT04116437) included 71 patients intolerant to prior BTK inhibitors (ibrutinib or acalabrutinib) with a median follow-up of 34.5 months [21]. The Chinese phase II multicenter study (NCT03206918) included 91 patients with R/R CLL/SLL and a median follow-up of 15.1 months [22]. All included trials were registered in clinical trial databases, ensuring methodological transparency. More details are presented in Table 1. 

### 3.3. Baseline Characteristics

Across the four included studies, the median age of patients receiving zanubrutinib ranged from 61 to 71 years, reflecting the older age distribution typical of patients with CLL. The proportion of female participants varied between 26.3% and 49.3%, with most studies reporting a male predominance. The majority of patients had an Eastern Cooperative Oncology Group (ECOG) performance status (PS) of 0–1, indicating good baseline functional status.

All studies enrolled patients with CLL or SLL, diagnosed according to established iwCLL or World Health Organization (WHO) criteria. In the phase III ALPINE trial, all patients had R/R CLL/SLL, with approximately 56% classified as Binet stage A/B and 44% as stage C [12]. Similarly, Xu et al. (2020) reported that 67.1% of patients were at Binet stage C at diagnosis, suggesting a higher disease burden [22]. In contrast, the Japanese study included both TN (73.7%) and R/R (26.3%) patients [20], while the trial by Shadman et al. (2025) specifically enrolled ibrutinib- or acalabrutinib-intolerant R/R patients (100%) [21].

Regarding prior therapy exposure, the proportion of patients who had received ≥2 previous lines of therapy ranged from 26% [12] to 49.5% [22]. Cytopenias at baseline were variably reported. Shadman et al. (2025) noted hemoglobin ≤ 110 g/L in 15.5%, platelet count ≤ 100 × 10^9^/L in 8.5%, and ANC ≤ 1.5 × 10^9^/L in 4.2% of patients [21], whereas Xu et al. (2020) reported elevated β_2_-microglobulin levels (>3.5 mg/L) in 72.5%, reflecting advanced disease [22].

Cytogenetic abnormalities were common across studies. The prevalence of del(17p) or TP53 mutations ranged from 0% [20] to 24.2% [22], while del(11q) was observed in 14–28% and del(13q) in up to 45% of patients. In the ALPINE trial, 22.9% had 17p deletion or TP53 mutation, and 27.8% harbored 11q deletion, with 17.1% showing a complex karyotype (≥3 abnormalities) [12].

Regarding immunoglobulin heavy chain variable region (IGHV) mutational status, the unmutated IGHV genotype predominated, observed in 56–73% of patients across trials. The presence of bulky disease, defined as lymph nodes ≥5 cm in the longest diameter, was reported in 44–45% of patients in the ALPINE and Xu et al. studies, respectively [12,22]. More details are presented in Table 2.

### 3.4. Quality Assessment

The methodological quality of the included studies was assessed using the JBI critical appraisal tools. The results are summarized in Table 3.

The study by Brown et al. (2023) [12], which was the sole RCT included, was judged to be of high quality. The study satisfied all key criteria for a robust RCT, including the use of true randomization, allocation concealment, similar baseline characteristics between groups, and an appropriate statistical analysis [12]. Furthermore, outcomes were measured reliably and identically across groups, and participants were analyzed according to their original assignment (intention-to-treat). The only notable limitation was the lack of blinding for the participants and those delivering the treatment, which is common in many interventional trials. However, the blinding of outcome assessors minimized the potential for detection bias.

The non-randomized studies were assessed using the JBI checklist. Overall, these three studies also demonstrated high methodological quality. All three studies clearly reported their inclusion criteria, used standard and valid methods to measure the condition of interest, and achieved complete inclusion of participants. Clinical information, demographics, and outcomes were reported clearly for all studies [20,21,22].

The primary area of uncertainty was for the item regarding the “clear reporting of the presenting sites/clinics’ demographic information,” which was rated as ‘Unclear’ for all three case series. Additionally, regarding the study by Izutse et al. (2025) [20], it was unclear whether the study consecutively included participants. Despite these minor uncertainties, all three case series employed appropriate statistical analysis and were deemed reliable for inclusion in the synthesis.

### 3.5. Outcomes

#### 3.5.1. Any-Grade Adverse Events

**Figure 2:**
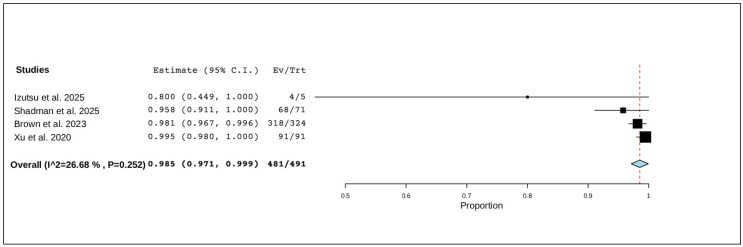
The rate of any-grade adverse events of R/R CLL [12,20,21,22].

Four studies, including a total of 491 patients, reported on any-grade adverse events. The pooled analysis indicated that nearly all patients experienced at least one adverse event, with a pooled proportion of 98.5% (95% CI, 97.1–99.9%), and no significant heterogeneity was observed across studies (I^2^ = 26.68%, *p* = 0.252). In total, 481 of 491 patients experienced an adverse event (Figure 2).

#### 3.5.2. Grade ≥ 3 Adverse Events

**Figure 3:**
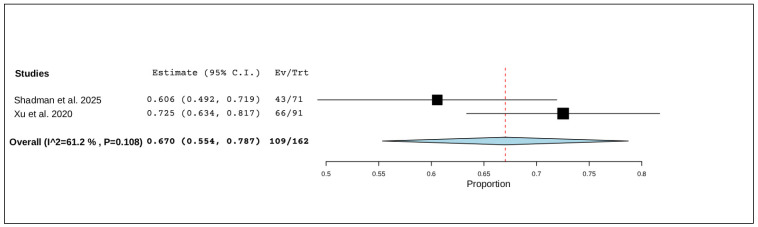
The rate of grade ≥3 adverse events of R/R CLL [21,22].

Two studies, including a total of 162 patients, reported on grade 3 or higher adverse events. The pooled analysis showed that these more severe events occurred in 67.0% of patients (95% CI, 55.4–78.7%), with moderate but non-significant heterogeneity (I^2^ = 61.2%, *p* = 0.108). In total, 109 of 162 patients experienced a grade ≥3 adverse event (Figure 3).

#### 3.5.3. Serious Adverse Events

**Figure 4:**
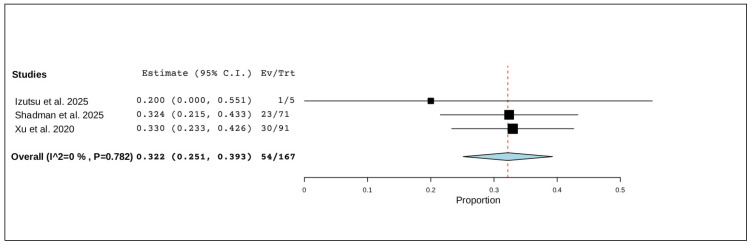
The rate of serious adverse events of R/R CLL [20,21,22].

Three studies, including a total of 167 patients, reported serious adverse events. The pooled proportion was 32.2% (95% CI, 25.1–39.3%), with no significant heterogeneity observed (I^2^ = 0%, *p* = 0.782). In total, 54 of 167 patients experienced a serious adverse event (Figure 4).

#### 3.5.4. Treatment Discontinuation Due to Adverse Events

**Figure 5:**
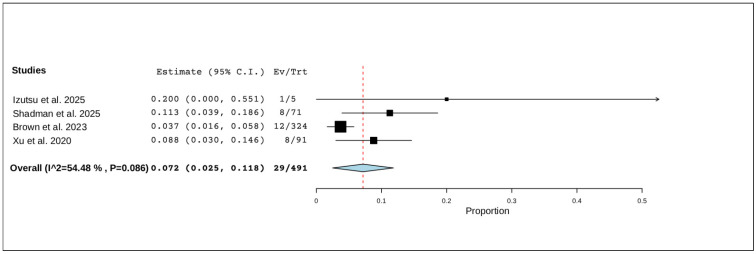
The rate of treatment discontinuation due to adverse events of R/R CLL [12,20,21,22].

Four studies, including a total of 491 patients, reported on treatment discontinuation due to adverse events. The pooled analysis found that 7.2% of patients (95% CI, 2.5–11.8%) discontinued treatment, with moderate but non-significant heterogeneity (I^2^ = 54.48%, *p* = 0.086). In total, 29 of 491 patients discontinued treatment (Figure 5).

#### 3.5.5. Adverse Event-Related Deaths

**Figure 6:**
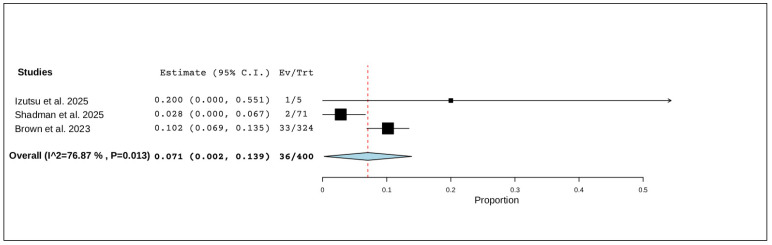
The rate of adverse event-related deaths of R/R CLL [12,20,21].

Three studies, including a total of 400 patients, reported on deaths related to adverse events. The pooled proportion was 7.1% (95% CI, 0.2–13.9%), with significant heterogeneity observed across studies (I^2^ = 76.87%, *p* = 0.013). In total, 36 of 400 patients died due to an adverse event (Figure 6).

### 3.6. Hematological Adverse Events

#### 3.6.1. Anemia

**Figure 7:**
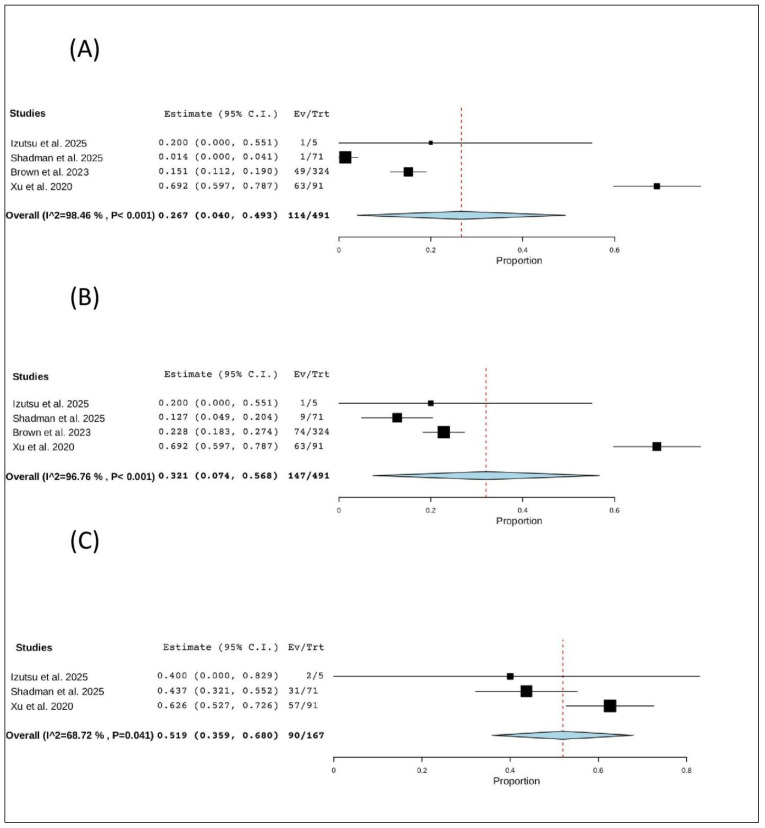
The rate of hematological adverse events of R/R CLL: (**A**) the rate of anemia; (**B**) the rate of neutropenia; (**C**) the rate of all bleeding events [12,20,21,22].

Four studies (491 patients) reported anemia. The pooled proportion was 26.7% (95% CI, 4.0–49.3%), with significant heterogeneity (I^2^ = 98.46%, *p* < 0.001). In total, 114 of 491 patients experienced anemia (Figure 7A).

#### 3.6.2. Neutropenia

Four studies (491 patients) reported neutropenia. The pooled proportion was 32.1% (95% CI, 7.4–56.8%), with significant heterogeneity (I^2^ = 96.76%, *p* < 0.001). In total, 147 of 491 patients experienced neutropenia (Figure 7B).

#### 3.6.3. All Bleeding Events

Three studies (167 patients) reported bleeding events. The pooled proportion was 51.9% (95% CI, 35.9–68.0%), with significant heterogeneity (I^2^ = 68.72%, *p* = 0.041). In total, 90 of 167 patients experienced a bleeding event (Figure 7C).

### 3.7. Non-Hematological Adverse Events

#### 3.7.1. All Cardiac Events

**Figure 8:**
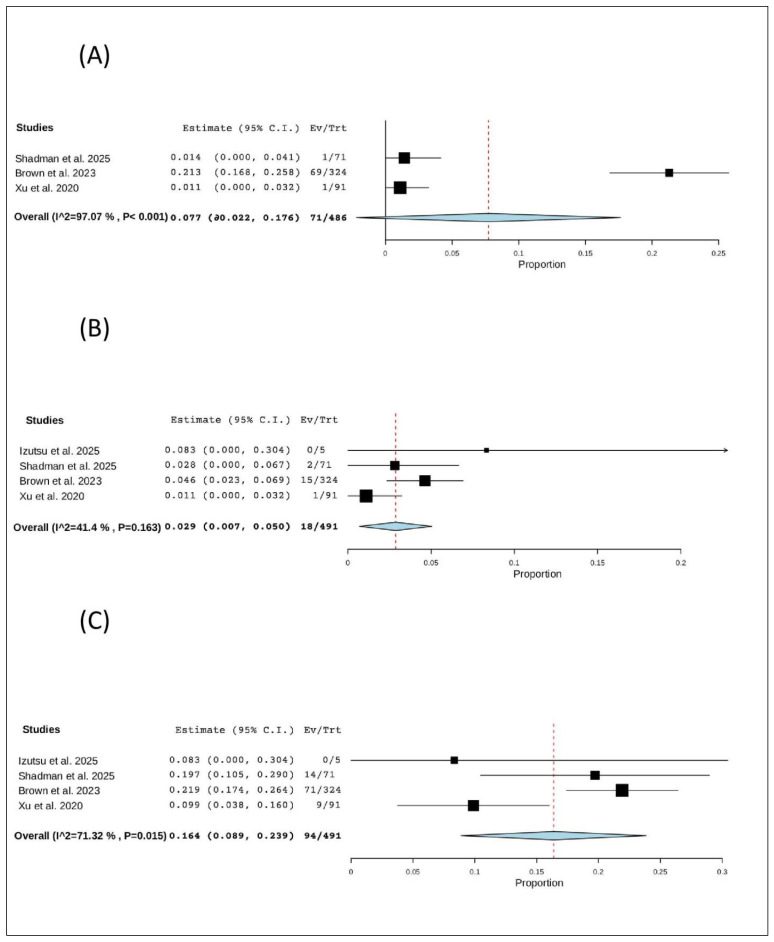
The rate of non-hematological adverse events of R/R CLL: (**A**) the rate of all cardiac events; (**B**) the rate of atrial fibrillation; (**C**) the rate of hypertension [12,20,21,22].

Three studies (486 patients) reported cardiac events. The pooled proportion was 7.7% (95% CI, −2.2–17.6%), with significant heterogeneity (I^2^ = 97.07%, *p* < 0.001). In total, 71 of 486 patients experienced a cardiac event (Figure 8A).

#### 3.7.2. Atrial Fibrillation

Four studies (491 patients) specifically reported atrial fibrillation. The pooled proportion was 2.9% (95% CI, 0.7–5.0%), with no significant heterogeneity (I^2^ = 41.4%, *p* = 0.163). In total, 18 of 491 patients experienced atrial fibrillation (Figure 8B).

#### 3.7.3. Hypertension

Four studies (491 patients) reported hypertension. The pooled proportion was 16.4% (95% CI, 8.9–23.9%), with significant heterogeneity (I^2^ = 71.32%, *p* = 0.015). In total, 94 of 491 patients experienced hypertension (Figure 8C).

#### 3.7.4. Pneumonia

**Figure 9:**
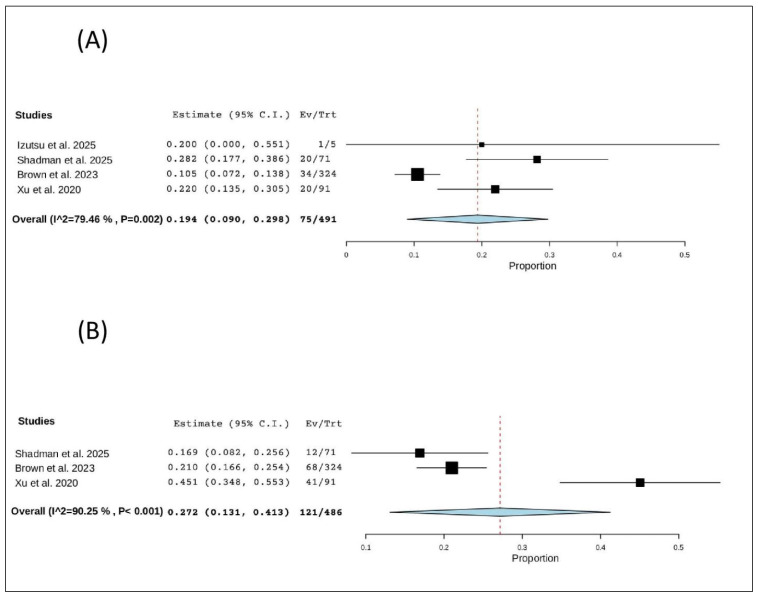
The rate of non-hematological adverse events of R/R CLL: (**A**) the rate of pneumonia; (**B**) the rate of upper respiratory tract infection [12,20,21,22].

Four studies (491 patients) reported pneumonia. The pooled proportion was 19.4% (95% CI, 9.0–29.8%), with significant heterogeneity (I^2^ = 79.46%, *p* = 0.002). In total, 75 of 491 patients experienced pneumonia (Figure 9A).

##### Upper Respiratory Tract Infection

Three studies (486 patients) reported upper respiratory tract infections. The pooled proportion was 27.2% (95% CI, 13.1–41.3%), with significant heterogeneity (I^2^ = 90.25%, *p* < 0.001). In total, 121 of 486 patients experienced an upper respiratory tract infection (Figure 9B).

## 4. Discussion

This systematic review and meta-analysis synthesized data from four clinical studies, including both randomized controlled and non-randomized trials, evaluating the safety of zanubrutinib (Brukinsa) in patients with R/R CLL and SLL. The pooled analysis provides an updated and comprehensive assessment of the safety profile of zanubrutinib in this population, integrating evidence from both controlled and real-world settings.

Overall, nearly all patients (98.5%) experienced at least one TEAE, which is expected in heavily pretreated populations. Despite this high incidence, severe and treatment-limiting toxicities were relatively uncommon. Grade ≥3 adverse events occurred in approximately two-thirds of patients (67.0%), while serious adverse events were reported in about one-third (32.2%). Importantly, only 7.2% of patients discontinued treatment due to toxicity, suggesting that zanubrutinib is generally well tolerated and that adverse events are not significant in most cases.

Hematological toxicities were the most frequently observed events, with neutropenia (32.1%) and anemia (26.7%) being the most common. These findings are consistent with prior reports of BTK inhibitors but appear less frequent than those observed with first-generation agents such as ibrutinib, which have demonstrated higher rates of hematologic suppression in comparable R/R CLL populations. Non-hematological adverse events included hypertension (16.4%), pneumonia (19.4%), and upper respiratory tract infections (27.2%), reflecting the known infection and cardiovascular risk profile associated with BTK inhibition.

Importantly, the incidence of atrial fibrillation, a major toxicity concern with ibrutinib, was notably low at 2.9% in this pooled analysis. This supports preclinical and clinical evidence suggesting that zanubrutinib’s higher selectivity for BTK and reduced off-target inhibition of tyrosine–protein kinase and epidermal growth factor receptor (EGFR) kinase resulted in fewer cardiac complications [7,11]. Similarly, all-grade bleeding events occurred in approximately 52% of patients, but most were mild to moderate, and major bleeding leading to discontinuation or death was rare.

Adverse event-related mortality was observed in 7.1% of patients, though substantial heterogeneity (I^2^ = 76.9%) suggests that this estimate varies according to study design, patient comorbidity burden, and disease stage. Notably, trials with broader inclusion criteria or those enrolling heavily pretreated patients tended to report higher mortality rates, highlighting the influence of baseline clinical factors. All of these findings point to zanubrutinib as a viable therapeutic choice for CLL/SLL patients who cannot tolerate ibrutinib or acalabrutinib.

Patients with CLL/SLL who suffered ibrutinib and acalabrutinib intolerance AEs were in line with the established safety profiles of both therapies in these patients [3,4]. At a median follow-up of 44 and 60 months, respectively, early trials of ibrutinib monotherapy in patients with R/R CLL/SLL (RESONATE) and treatment-naïve CLL/SLL (RESONATE-2) revealed that 12% and 28% of patients discontinued due to toxicity [24,25]. Pneumonia, anemia, thrombocytopenia, diarrhea, and anal incontinence in RESONATE and atrial fibrillation, palpitations, and pneumonia in RESONATE-2 were the most frequent toxicities that resulted in stopping ibrutinib [24,25]. Furthermore, in RESONATE and RESONATE-2, there were reports of hypertension in 20% and 26%, atrial fibrillation in 11% and 16%, and significant bleeding in 6% and 11%, respectively [24,25]. At a median follow-up of 40.9 months, 15% of patients treated with acalabrutinib experienced treatment discontinuation due to AEs in a trial comparing acalabrutinib and ibrutinib in patients with R/R CLL; the most frequent AEs resulting in treatment discontinuation were infections, cytopenia, and second primary malignancies [3]. Nine percent of patients on acalabrutinib experienced both hypertension and atrial fibrillation/flutter. The most frequent ibrutinib intolerance AEs in the BGB-3111-215 study were fatigue, atrial fibrillation, rash, arthralgia, and stomatitis, while the most frequent acalabrutinib intolerance AEs were headache, rash, diarrhea, hemorrhage, myalgia, and arthralgia.

Regardless of whether patients were intolerant of ibrutinib or acalabrutinib, the percentage of therapy termination due to TEAEs with zanubrutinib was low (11% at a median follow-up of 34.5 months). It has already been demonstrated that zanubrutinib treatment causes fewer cardiovascular adverse events than ibrutinib. Although there was no discernible difference in the rate of hypertension (24% vs. 23%), patients with R/R CLL who received zanubrutinib vs. ibrutinib had fewer AEs that resulted in treatment discontinuation (15% vs. 22%) and fewer instances of atrial fibrillation or flutter (5% vs. 13%) [12].

Atrial fibrillation (2 of 16 patients), diarrhea (5 of 7), rash (3 of 7), bleeding (5 of 6), and arthralgia (2 of 7) were among the 27 ibrutinib-intolerance adverse events (AEs) that recurred in 24 out of 60 patients (40%) in a study of acalabrutinib in patients with R/R CLL who were intolerant to the drug. The study had a median follow-up of 35.6 months (range, 1.1–47.4) [26]. Compared to ibrutinib, one incident (an elevated liver function test) happened at a higher grade with acalabrutinib. In this case, intolerance adverse events (AEs) recurred in 46% and 30% of patients who were intolerant of ibrutinib and acalabrutinib, respectively, although not at a higher grade. Just one patient stopped taking their medication because of a recurrent ibrutinib-intolerance adverse event, and two patients stopped taking their medication because of a recurrent acalabrutinib-intolerance adverse event. Zanubrutinib’s reduced binding to off-target kinases and increased BTK selectivity may be responsible for its lower toxicity profile when compared to ibrutinib [11]. Only 7 off-target kinases were inhibited by zanubrutinib when tested against a panel of 370 kinases; in contrast, ibrutinib inhibited 17, acalabrutinib inhibited 15, and M27, a metabolite of acalabrutinib, inhibited 23. Additionally, zanubrutinib was ≥33 times more effective than acalabrutinib in inhibiting BTK [12].

Several safety outcomes observed with first-generation BTK inhibitors, particularly ibrutinib, have been linked to off-target kinase inhibition. Ibrutinib’s covalent binding to BTK also affects other kinases such as TEC, EGFR, SRC family kinases, and C-terminal Src kinase (CSK), which are involved in cardiac conduction and platelet function, contributing mechanistically to cardiovascular toxicities like atrial fibrillation, hypertension, and bleeding events seen in clinical practice and real-world studies [27]. Off-target inhibition of these kinases may disrupt phosphoinositide 3-kinase–Akt signaling and cardiac electrophysiology, thereby increasing pro-arrhythmic risk and adverse hematologic effects [27].

In contrast, next-generation BTK inhibitors such as zanubrutinib were designed with increased selectivity for BTK and reduced inhibition of many of these off-target kinases [28]. This pharmacologic refinement has been associated with improved safety outcomes in clinical trials and pooled safety analyses, including a lower incidence of atrial fibrillation and other cardiovascular toxicities compared with ibrutinib [29]. The enhanced selectivity of zanubrutinib likely limits interference with kinases implicated in arrhythmogenesis and platelet activation, providing a biologically plausible explanation for the more favorable safety profile observed in our meta-analysis [11].

Taken together, the findings of this review indicate that zanubrutinib maintains a favorable safety profile across diverse populations and geographic regions. Compared with first-generation BTK inhibitors, it appears to reduce the incidence of key off-target toxicities, particularly atrial fibrillation and severe bleeding, while maintaining manageable hematologic and infectious adverse events.

### 4.1. Strengths and Limitations

This review offers several strengths. It is the first systematic review and meta-analysis specifically focused on the safety of zanubrutinib in patients with R/R CLL/SLL, following PRISMA and Cochrane guidelines with a pre-registered PROSPERO protocol. Comprehensive database searches, rigorous risk-of-bias assessments using validated JBI tools, and the use of random-effects modeling enhanced the robustness and generalizability of the findings. Moreover, inclusion of data from a large phase III RCT alongside smaller phase I/II studies allowed for a balanced synthesis of evidence from both controlled and real-world settings.

This review addresses a timely and clinically relevant topic, as zanubrutinib, a next-generation BTK inhibitor, is increasingly used in patients with relapsed or R/R CLL, yet comprehensive data on its safety profile across studies remain limited. This review responds to an urgent clinical need for consolidated evidence on its tolerability. It fills an important evidence gap, since while the efficacy of zanubrutinib has been well-documented, a systematic synthesis of its adverse event spectrum, particularly in heavily pretreated or high-risk populations, has not been comprehensively performed. Additionally, the findings will support clinical decision-making by helping clinicians better understand pooled safety outcomes, thereby improving treatment selection, patient monitoring, and adverse event management. Furthermore, the review provides relevance for safety assessment, serving as a benchmark for future head-to-head evaluations of BTK inhibitors such as ibrutinib and acalabrutinib, ultimately guiding personalized therapy. Lastly, it offers potential regulatory and pharmacovigilance implications, as the integration of real-world and clinical trial safety data may inform clinicians, regulators, and policymakers in refining treatment guidelines and strengthening post-marketing safety surveillance.

On the other hand, this review has several limitations that should be considered when interpreting the findings. First, the number of included studies was limited to four RCTs and the total pooled sample size (508 patients), reflecting the currently available evidence on zanubrutinib in CLL/SLL. The restricted number of trials may reduce the statistical power of the pooled analyses and limit the precision of the effect estimates. In addition, the small evidence base may affect the generalizability of the findings across broader patient populations and clinical settings. Therefore, further large-scale, well-designed RCTs are warranted to validate the present results and provide more robust evidence regarding the safety profile of zanubrutinib.

In addition to the limited number of included studies and small sample sizes, several other factors should be considered when interpreting the results. First, many of the included trials had short follow-up periods, which may have underestimated the incidence of long-term or delayed toxicities. Second, there was inconsistent reporting and assessment of specific adverse events, including infections, bleeding, and cardiovascular events, across trials. These limitations highlight the need for cautious interpretation of the pooled safety data and the importance of longer-term, standardized safety monitoring in future studies.

Additionally, despite the inclusion of one RCT, the remaining studies were open-label single-arm or multicenter trials, which may introduce selection and reporting bias. Third, heterogeneity was significant for several outcomes (e.g., hematologic events, infections, hypertension), likely to reflect variations in study design, population characteristics, follow-up duration, and adverse event grading systems. Fourth, the analysis was limited to published data; individual patient-level data were not available to allow for subgroup analyses (e.g., by age, prior BTK exposure, or cytogenetic risk). Finally, long-term toxicities such as secondary malignancies, late cardiac events, or cumulative infection risk could not be fully assessed due to limited follow-up in most included trials.

### 4.2. Clinical Implications

The findings of this meta-analysis have important clinical implications for the management of patients with R/R CLL. Zanubrutinib demonstrates a significant and predictable safety profile with a low rate of treatment discontinuation due to adverse events, supporting its use as a preferred next-generation BTK inhibitor in this setting. The markedly lower incidence of atrial fibrillation compared with ibrutinib is particularly relevant for elderly patients or those with preexisting cardiovascular comorbidities. Additionally, the hematologic toxicity profile, though notable, is largely manageable through dose modification or supportive care. Clinicians should remain vigilant for infections and hypertension, which continue to represent relevant AEs with BTK inhibition. Overall, these results reinforce zanubrutinib as a well-tolerated long-term therapy option for patients with R/R CLL, particularly those who are intolerant to prior BTK inhibitors.

## 5. Conclusions

This systematic review and meta-analysis demonstrated that zanubrutinib (Brukinsa) exhibits a favorable safety and tolerability profile in patients with R/R CLL. While nearly all patients experience at least one AE, severe or treatment-limiting toxicities remain infrequent, and treatment discontinuations due to adverse events are low. The low incidence of atrial fibrillation and significant rates of hematologic and infectious AEs support zanubrutinib as a safer alternative to first-generation BTK inhibitors. Future research with larger RCTs and long-term follow-up is warranted to further define its comparative safety, particularly in real-world populations and patients with high-risk cytogenetic features.

## Figures and Tables

**Table 1:** Summary of the included studies [12,20,21,22].

Study ID	Type of Study	Country	Duration	Trial Registration	Type of Patients	Diagnostic Criteria	Zanubrutinib Regimen (Dose, Route of Administration, Regimen)	No. of Patients in the Zanubrutinib Group	Follow-Up, Months, Median (Range)
Izutse et al. 2025 [20]	Phase I/II multicenter, open-label study	Japan	From 30 January 2020 to 31 October 2022	NCT04172246	Japanese adults (age ≥ 20 years) with TN or R/R CLL/SLL or WM. Also included patients with other B-cell malignancies in Part 1 and an R/R MCL cohort in Part 2 for safety analysis.	CLL/SLL: 2018 iwCLL guidelines; SLL: Lugano classification; WM: 6th International Workshop on WM criteria	Dose: 160 mg twice daily Route: orally Regimen: continuously until disease progression, unacceptable toxicity, or other discontinuation criteria	CLL/SLL: 19 (TN: 14, R/R: 5)	27.9
Shadman et al. 2025 [21]	Phase II, single-arm, open-label clinical trial	United States	Data cut-off: 1 May 2024	NCT04116437	Patients with CLL or SLL intolerant of prior ibrutinib and/or acalabrutinib therapy.	iwCLL criteria	Dose: 160 mg twice daily or 320 mg once daily Route: Oral (implied) Regimen: Continuous dosing; patients/investigators selected a regimen and could not switch	71	34.5 (0.1–51.1)
Brown et al. 2023 [12]	Phase III, randomized, open-label, active-controlled, multinational, clinical trial	Multinational (15 countries across North America, Europe, and Asia-Pacific)	From 1 November 2018 to 14 December 2020	NCT03734016	Relapsed or refractory CLL or SLL	iwCLL criteria	Dose: 160 mg twice daily Route: orally Regimen: until disease progression or unacceptable toxicity	327 patients in the zanubrutinib group (intention-to-treat population)	29.6
Xu et al. 2020 [22]	Phase II, single-arm, multicenter study	China	From 9 March to 14 December 2017	CTR20160890 (7 December 2016) NCT03206918 (2 July 2017)	R/R CLL or SLL	CLL: iwCLL guidelines (2008); SLL: WHO classification (2008), histologically confirmed by central pathologic review	160 mg orally twice daily in 28-day cycles until disease progression or intolerance	91	15.1 (0.8–21.2)

Abbreviations: TN: treatment-naïve, R/R: relapsed/refractory, CLL: chronic lymphocytic leukemia, SLL: small lymphocytic lymphoma, WM: Waldenström macroglobulinemia, MCL: mantle cell lymphoma, iwCLL: International Workshop on Chronic Lymphocytic Leukemia, WHO: World Health Organization.

**Table 2:** Baseline characteristics of the included patients [12,20,21,22].

Study ID	Age, Median (Range)	Female *n* (%)	ECOG PS, No. (%)	Type of Cancer No. (%)	Binet Stage at CLL Diagnosis, *n* (%)	Prior Treatment Status No. (%)	Cytopenia at Baseline, No. (%)	Chromosomal Mutation Status, No. (%)	IGHV Mutational Status—No. (%)	Bulky Disease—No. (%)
Izutse et al. 2025 [20]	71.0 (38–77)	5 (26.3)	0: 17 (89.5) 1: 2 (10.5)	CLL/SLL: 19 (100)	NR	TN: 14 (73.7%) R/R: 5 (26.3%) R/R CLL/SLL (*n* = 5)	NR	CLL/SLL: del(17p): 0 (0)	Mutated: 12 (63.2) Unmutated: 7 (36.8)	NR
Shadman et al. 2025 [21]	71 (49–91)	35 (49.3)	0:44 (62) 1: 25 (35.2) 2: 2 (2.8)	CLL: 63 (88.7) SLL: 8 (11.3)	A: 12 (16.9) B: 6 (8.5) C: 4 (5.6) Unknown: 41 (57.7)	R/R: 71 (100%)	Hemoglobin ≤ 110 g/L: 11 (15.5) Platelet count ≤ 100 × 10^9^/L: 6 (8.5) ANC ≤ 1.5 × 10^9^/L: 3 (4.2)	del(11q): 10 (14.1) del(17p): 6 (8.5) del(13q): 17 (23.9) TP53 mutation: 16 (22.5)	Unmutated: 13 (18.3) Mutated: 12 (16.9) Unknown/Missing: 46 (64.8)	Largest diameter < 5 cm: 49 (69) Largest diameter > 5 cm: 10 (14.1) No measurable disease: 12 (16.9)
Brown et al. 2023 [12]	67 (35–90) ≥65 and <75: 127 (38.8) ≥75: 74 (22.6)	114 (34.9)	≥1: 198 (60.6)	CLL or SLL: 327 (100)	A/B: 182 (55.7) C: 145 (44.3)	1 line: Zanubrutinib 192 (58.7) 2 lines: 86 (26.3) 3 lines: 25 (7.6) 3 lines: Zanubrutinib 24 (7.3)	NR	17p deletion and/or TP53 mutation: 75 (22.9) 11q deletion: Zanubrutinib 91 (27.8%), I Complex karyotype (≥3 abnormalities): 56 (17.1)	Unmutated: 239 (73.1) Mutated: 79 (24.2) Missing:9 (2.8)	Yes (tumor ≥ 5 cm): 145 (44.3)
Xu et al. 2020 [22]	61 (35–87)	39 (42.9)	0/1: 88 (96.7) 2: 3 (3.3)	CLL: 82 (90.1) SLL: 9 (9.9)	A/B: 27 (32.9) C: 55 (67.1)	Refractory to last systemic therapy: 72 (79.1) non-refractory: 19 (20.9) ≥2 prior lines: 45 (49.5)	Present: 66 (72.5) had a baseline serum β2-microglobulin level > 3.5 mg/L Absent: 25 (27.5) had a baseline absolute neutrophil count < 1.5 × 10^9^/L.	del(17p) or TP53 mutation: 22 (24.2) del(11q): 20 (22.0) del(13q): 41 (45.1) Trisomy 12: 21 (23.1)	Unmutated: 51 (56.0) Mutated: 23 (25.3) Not available: 17 (18.7)	Present (≥1 lesion with LDI ≥ 5 cm): 40 (44.0) Absent: 51 (56.0)

Abbreviations: CLL: chronic lymphocytic leukemia, SLL: small lymphocytic lymphoma.

**Table 3:** Quality assessment of the randomized and non-randomized clinical trials using the JBI tool [12,20,21,22].

Study ID	Brown et al. 2023 [12]	Study ID	Izutse et al. 2025 [20]	Shadman et al. 2025 [21]	Xu et al. 2020 [22]
Was true randomization used for assignment of participants to treatment groups?	Yes	1. Were there clear criteria for inclusion in the case series?	Yes	Yes	Yes
Was allocation to treatment groups concealed?	Yes	2. Was the condition measured in a standard, reliable way for all participants included in the case series?	Yes	Yes	Yes
Were treatment groups similar at the baseline?	Yes	3. Were valid methods used for identification of the condition for all participants included in the case series?	Yes	Yes	Yes
Were participants blind to treatment assignment?	No	4. Did the case series have consecutive inclusion of participants?	Unclear	Yes	Yes
Were those delivering treatment blind to treatment assignment?	No	5. Did the case series have complete inclusion of participants?	Yes	Yes	Yes
Were outcomes assessors blind to treatment assignment?	Yes	6. Was there clear reporting of the demographics of the participants included in the study?	Yes	Yes	Yes
Were treatment groups treated identically, other than the intervention of interest?	Yes	7. Was there clear reporting of clinical information of the participants?	Yes	Yes	Yes
Was follow-up complete, and if not, were differences between groups in terms of their follow-up adequately described and analyzed?	Yes	8. Were the outcomes or follow-up results of cases clearly reported?	Yes	Yes	Yes
Were participants analyzed in the groups to which they were randomized?	Yes				
Were outcomes measured in the same way for treatment groups?	Yes	9. Was there clear reporting of the presenting sites’/clinics’ demographic information?	Unclear	Unclear	Unclear
Were outcomes measured in a reliable way?	Yes				
Was appropriate statistical analysis used?	Yes	10. Was statistical analysis appropriate?	Yes	Yes	Yes
Was the trial design appropriate, and were any deviations from the standard RCT design (individual randomization, parallel groups) accounted for in the conduct and analysis of the trial?	Yes				

## Data Availability

No new data were created or analyzed in this study. Data sharing is not applicable.
